# Differential regulation of nitric oxide synthase function in aorta and tail artery from 5/6 nephrectomized rats

**DOI:** 10.1002/phy2.145

**Published:** 2013-11-05

**Authors:** Frank T Spradley, John J White, William D Paulson, David M Pollock, Jennifer S Pollock

**Affiliations:** 1 Section of Experimental Medicine, Georgia Regents UniversityAugusta, Georgia; 2 Department of Medicine, Georgia Regents UniversityAugusta, Georgia; 3 Charlie Norwood VA Medical Center, Georgia Regents UniversityAugusta, Georgia

**Keywords:** Aorta, hypertension, L-NAME, nephrectomy, NOS, tail artery, vascular reactivity

## Abstract

Chronic renal failure (CRF) is associated with hypertension and concomitant endothelial dysfunction, enhanced vasoconstriction, and nitric oxide synthase (NOS) dysfunction. Vascular function in patients is assessed in peripheral extremity arteries like the finger arteries, whereas animal studies often use the centrally located aorta. Therefore, we examined whether peripheral tail artery and aortic NOS function are differentially regulated by blood pressure in rats with CRF. Using wire myography, arterial function was assessed in 16-week-old Sprague-Dawley rats that were subjected to 5/6 nephrectomy (Nx; arterial ligation model) 8 weeks earlier or non-Nx (control) rats. In aortas from Nx rats, endothelial-dependent vasorelaxation response to acetylcholine (ACh) was blunted and there was enhancement of phenylephrine (PE)-mediated vasoconstriction. Inversely, tail arteries from Nx rats had no change in endothelial function and reduced response to PE. Studies where arterial segments were incubated with the nonspecific NOS inhibitor, L-NAME, showed that Nx reduced NOS function in the aorta but increased NOS function in tail artery for both ACh and PE responses. Furthermore, the observed alterations in NOS function in both aorta and tail artery were abolished when mean arterial blood pressure, as assessed by telemetry, was maintained at normal levels in the 5/6 Nx rats using triple therapy: hydralazine (30 mg/kg per day), hydrochlorothiazide (10 mg/kg per day), and reserpine (0.5 mg/kg per day). In conclusion, differential changes of NOS function in central versus peripheral arteries in CRF are dependent upon hypertension.

## Introduction

Chronic renal failure (CRF) is associated with distinct changes in vascular function (Bolton et al. 2001). Notably endothelial dysfunction, which is classically detected as a blunted acetylcholine (ACh)-induced vasorelaxation response, is a consistent finding as demonstrated in omental arteries from patients with end-stage renal disease (Annuk et al. 2001; Luksha et al. 2012). Furthermore, omental arteries from patients with kidney disease have an augmented vasoconstriction response to the *α*_1_-adrenoceptor agonist phenylephrine (PE) (Cruz-Dominguez et al. 2008). These changes in vascular function have been attributed to several risk factors, not the least of which is hypertension (Guerin et al. 2008). It is well established that hypertension is an independent risk factor for cardiovascular disease (Rehman and Schiffrin 2010). Hypertension is closely linked to mechanisms leading to dysfunctional regulation of nitric oxide synthase (NOS) (Sasser et al. 2004). NOS, particularly the endothelial isoform (eNOS, NOS3), is a critical component for endothelial function and is antiatherogenic (Kuhlencordt et al. 2001). Atherosclerosis is the leading cause of death in patients with progressive kidney disease (Balla et al. 2013).

The 5/6 nephrectomized (Nx) rat is an established model of CRF having a significant reduction in renal mass along with concomitant hypertension (Pollock et al. 1993). Mechanistic studies assessing vascular function in this model have focused on aortic dysfunction in CRF, which is closely linked with NOS dysfunction (Vaziri 2001). Moreover, there is a reduction in NOS3 expression and NO bioavailability in the thoracic aorta from 5/6 Nx rats (Hasdan et al. 2002; Toba et al. 2011). However, similar to the studies above in human omental arteries, these aortic studies were conducted ex vivo and required dissection of the arterial segments. In humans, assessment of more accessible peripheral arteries of the extremities without requiring dissection is a more feasible measure to detect cardiovascular disease outcomes. For example, those patients with coronary artery disease have detectable reduction in blood flow in finger arteries (Qureshi et al. 2002). Assessment of finger artery blood flow using peripheral arterial tonometry is capable of detecting endothelial dysfunction in patients with hypertension, hyperlipidemia, and diabetes mellitus (Kuvin et al. 2003). To model an extremity artery in rodents, function of the proximal section of the rat tail artery, which is considered a medium-sized artery has been studied (Bessa et al. 2011). Intriguingly, it has been demonstrated that the tail artery has a blunted response to PE-induced constriction in the setting of 5/6 Nx (Brymora et al. 2007).

The mechanisms explaining this blunted constrictive response in the tail artery from 5/6 Nx rats are not known. Vascular biology studies demonstrate that NOS is important in blunting the vasoconstriction provoked by PE (Malmstrom et al. 2001). Therefore, we hypothesized that the differential functional responses of aorta and tail artery from 5/6 Nx rats depend on reduced and increased NOS function, respectively. We found a reduction in NOS-mediated vasorelaxation in aortas but an increase in this response in tail arteries from 5/6 Nx rats. Both of these differing responses required the development of hypertension in this model of CRF.

## Methods

### Animals and 5/6 Nx protocol

Seven-week-old male Sprague-Dawley rats were purchased from Harlan Laboratories (Indianapolis, IN). All rats were provided with food (Teklad 8604; Harlan Laboratories) and water ad libitum for the duration of all studies. Animal use protocols were preapproved by the Institutional Animal Care and Use Committee at Georgia Regents University. At 8 weeks old, rats underwent 5/6 Nx as described previously (Pollock et al. 1993). Briefly, rats were anesthetized with isoflurane (Aerrane; Baxter, Deerfield, IL) and a midline incision made to facilitate removal of the right kidney and ligation of two primary branches of the left renal artery. All rats were housed individually.

### Telemetry blood pressure measurements

On the day of 5/6 Nx surgery, rats were also implanted with telemetry transmitters (Data Sciences, Inc, St. Louis, MO) for determination of mean arterial blood pressure in conscious rats as described previously (D'Angelo et al. 2006). Telemetry measurements were collected every 10th minute and data expressed as 24 h means.

### Antihypertensive treatment

Blood pressure was controlled with triple therapy (TTx) treatment for the duration of 5/6 Nx (8 weeks) as previously described by our laboratory (Kang et al. 2011). The TTx regimen consisted of hydralazine (30 mg/kg per day), hydrochlorothiazide (10 mg/kg per day), and reserpine (0.5 mg/kg per day). All chemicals were purchased from Sigma unless otherwise noted. TTx was provided in drinking water ad libitum for the entire duration of the study. Vehicle treatment consisted of tap water. Successful reduction in blood pressure with TTx in 5/6 Nx was monitored via telemetry.

### Artery isolation and vascular reactivity protocol

Thoracic aortas and proximal tail arteries were isolated and prepared for vascular function studies as described previously (Spradley et al. 2012). Nx and control rats were anesthetized using 50 mg/kg pentobarbital sodium (Nembutal: Abbott Laboratories, North Chicago, IL). Aortas were mounted on pins and tail arteries on chucks for arterial wire myography (Danish Myo Technology A/S, Denmark). Cumulative concentration–response curves were generated to assess endothelial-dependent vasorelaxation with ACh (1 × 10^−9^ to 3 × 10^−5^ mol/L) following constriction with a submaximal dose of (PE; 10^−7^). Endothelial-independent vasorelaxation with sodium nitroprusside (SNP; 1 × 10^−10^ to 3 × 10^−5^ mol/L) was assessed in the same vessel segment, which was constricted with PE. Vasoconstriction was assessed with PE (1 × 10^−9^ to 3 × 10^−5^ mol/L) followed by KCl (8 to 100 mmol/L) in the same vessel segment. PE and KCl responses were normalized against the maximum response to KCl. To examine the maximum response to KCl, % increase in force was analyzed by the following equation: ((response to vasoconstrictor – baseline prior to constriction)/baseline prior to constriction) ×100. To assess NOS function, vessel segments were incubated ± L-NAME (L-N^G^-Nitroarginine methyl ester) (nonspecific NOS-inhibitor; 100 *μ*mol/L) for 15 min prior to ACh and PE.

### Statistical analysis

All data are expressed as mean ± standard error of the mean. Percent maximum response and sensitivity (logEC_50_ or EC_50_) to the vasoactive agonists in the vascular reactivity experiments were calculated using GraphPad Prism (La Jolla, CA). Statistical significance of vascular reactivity data was assessed with a Student's *t*-test, whereas the telemetry data were assessed using two-way analysis of variance (ANOVA) for repeated measures combined with a Bonferroni posttest to compare replicate means (GraphPad Prism).

## Results

### Aortic reactivity following 8 weeks of 5/6 Nx

**Figure 1:**
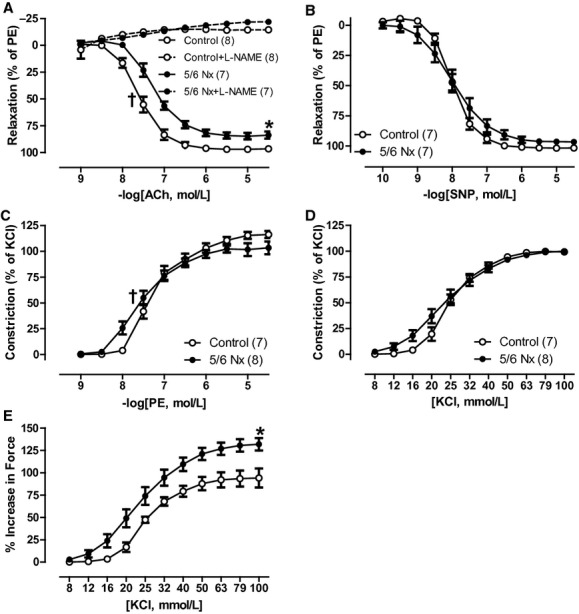
Response of aortas isolated from control and 5/6 nephrectomized (Nx) rats to (A) acetylcholine (ACh), (B) sodium nitroprusside (SNP), and (C) phenylephrine (PE). Response to KCl analyzed as constriction (% of KCl) to assess EC_50_ (D) and % increase in force to examine maximum constriction response (E). Dependence of ACh-induced relaxation on NOS was assessed in the presence of the nonspecific NOS inhibitor L-NAME in aortas isolated from control and 5/6 Nx (A) rats. The number of rats is in parentheses. *P < 0.05 versus control for % maximum response; ^†^*P* < 0.05 for logEC_50_ versus control.

Aortas isolated from 5/6 Nx rats displayed reduced maximum responsiveness (E_max_) (5/6 Nx: 83 ± 3% vs. control: 96 ± 2%, *P* < 0.05) and sensitivity (logEC_50_) (5/6 Nx: −7.0 ± 0.1 mol/L vs. control: −7.5 ± 0.1 mol/L, *P* < 0.05) to ACh compared with control rats (Fig. 1A). In contrast, there were no differences detected in E_max_ (5/6 Nx: 96 ± 2% vs. control: 101 ± 0.4%) or logEC_50_ (5/6 Nx: −8.1 ± 0.2 mol/L vs. control: −8.0 ± 0.1 mol/L) aortic responses to SNP (Fig. 1B).

Aortas from 5/6 Nx rats had no change in the E_max_ response to PE (5/6 Nx: 103 ± 6% vs. control: 116 ± 3%), although there was a slight but statistically significant increase in sensitivity to PE (logEC_50_: 5/6 Nx: −7.6 ± 0.07mol/L vs. control: −7.3 ± 0.05 mol/L, *P* < 0.05) (Fig. 1C). This increased aortic sensitivity to PE in 5/6 Nx rats occurred without alterations in sensitivity to KCl (EC_50_: 5/6 Nx: 22.9 ± 1.7 mmol/L vs. control: 24.6 ± 1.1 mmol/L) (Fig. 1D) but with an increase in the maximum response to KCl (% increase in force: 5/6 Nx: 137.4 ± 10.0% vs. control: 107 ± 8.7%, *P* < 0.05) (Fig. 1E).

### Aortic NOS function following 8 weeks of 5/6 Nx

The ACh-induced aortic relaxation was totally dependent on NOS function in control rats and 5/6 Nx rats indicating that the reduced ACh response in aortas from Nx rats was due to loss of NOS function (Fig. 1A).

As for PE constriction, L-NAME treatment of aortas from control rats significantly enhanced both sensitivity (logEC_50_: +L-NAME: −7.8 ± 0.05 mol/L vs. −L-NAME: −7.3 ± 0.05 mol/L, *P* < 0.05) and E_max_ (+L-NAME: 130 ± 2% vs. −L-NAME: 116 ± 3%, *P* < 0.05), whereas, in 5/6 Nx rats, L-NAME did not alter logEC_50_ to PE (+L-NAME: −7.8 ± 0.09 mol/L vs. −L-NAME: −7.6 ± 0.07 mol/L), but increased E_max_ (+L-NAME: 119 ± 1% vs. −L-NAME: 106 ± 6%, *P* < 0.05).

### Tail artery reactivity following 8 weeks of 5/6 Nx

**Figure 2:**
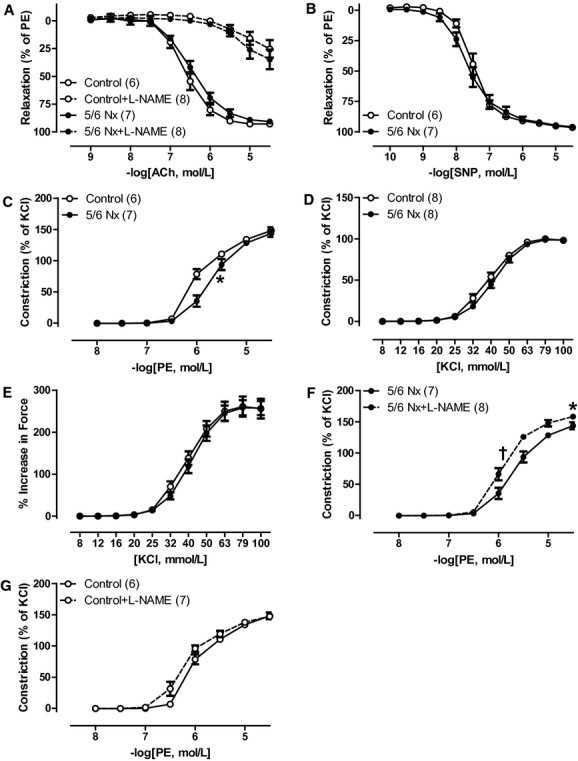
Response of tail arteries isolated from control and 5/6 nephrectomized (Nx) rats to (A) acetylcholine (ACh), (B) sodium nitroprusside (SNP), and (C) phenylephrine (PE). Response to KCl analyzed as constriction (% of KCl) to assess EC_50_ (D) and % increase in force to examine maximum constriction response (E). NOS buffering capacity to PE-induced constriction was assessed in the presence of the nonspecific NOS inhibitor L-NAME in tail arteries from 5/6 Nx (F) and control (G) rats. The number of rats is in parentheses. *P < 0.05 versus control for % maximum response; ^†^*P* < 0.05 for logEC_50_ (or EC_50_ for KCl) versus control.

No differences were detected for the response to ACh in tail arteries from control or 5/6 Nx rats (E_max_: 5/6 Nx: 90 ± 2% vs. control: 92 ± 1%; logEC_50_: 5/6 Nx: −6.4 ± 0.1 mol/L vs. control: −6.5 ± 0.1 mol/L) (Fig. 2A). Similarly, the response to SNP was not altered in tail arteries from 5/6 Nx rats compared with controls (E_max_: 5/6 Nx: 95.6 ± 1.2% vs. control: 96.2 ± 1.2%; logEC_50_: 5/6 Nx: −7.5 ± 0.1 mol/L vs. control: −7.4 ± 0.09 mol/L) (Fig. 2B).

Tail arteries from 5/6 Nx rats had blunted sensitivity to PE (logEC_50_: 5/6 Nx: −5.7 ± 0.06 mol/L vs. control: −6.0 ± 0.07 mol/L, *P* < 0.05), whereas E_max_ was similar between groups (5/6 Nx: 151 ± 6.9% vs. control: 148.4 ± 5.3%) (Fig. 2C). This reduced sensitivity was not associated with changes in KCl sensitivity (EC_50_: 5/6 Nx: 41.3 ± 1.2 mmol/L vs. control: 38.0 ± 1.3 mmol/L) (Fig. 2D) or maximum response (% increase in force: 5/6 Nx: 257.6 ± 16.8 vs. control: 256.5 ± 23.6) (Fig. 2E).

### Tail artery NOS function following 8 weeks of 5/6 Nx

L-NAME blunted the E_max_ and logEC_50_ responses to ACh similarly in control and 5/6 Nx rats (Fig. 2A). In contrast, L-NAME increased E_max_ and logEC_50_ to PE in tail arteries from 5/6 Nx rats (Fig. 2F), but did not alter E_max_ and logEC_50_ to PE in tail arteries from control rats (Fig. 2G). These data indicate an enhancement of NOS-mediated buffering of vasoconstriction in tail arteries from rats with CRF.

### Effect of antihypertensive treatment on aorta and tail artery NOS function following 8 weeks of 5/6 Nx rats

**Figure 3:**
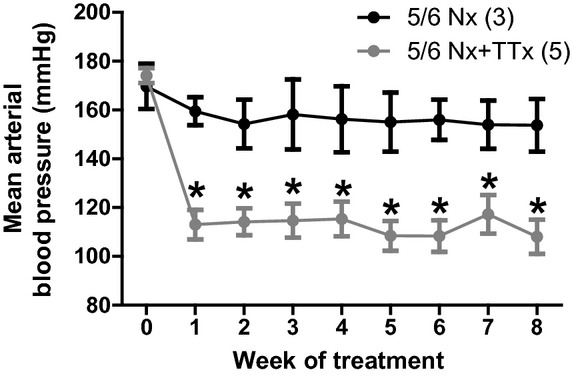
Mean arterial pressure (MAP) in 5/6 nephrectomized (Nx) rats treated ± triple therapy (TTx) for duration of Nx. The number of rats is in parentheses. *P < 0.05 versus untreated rats.

**Figure 4:**
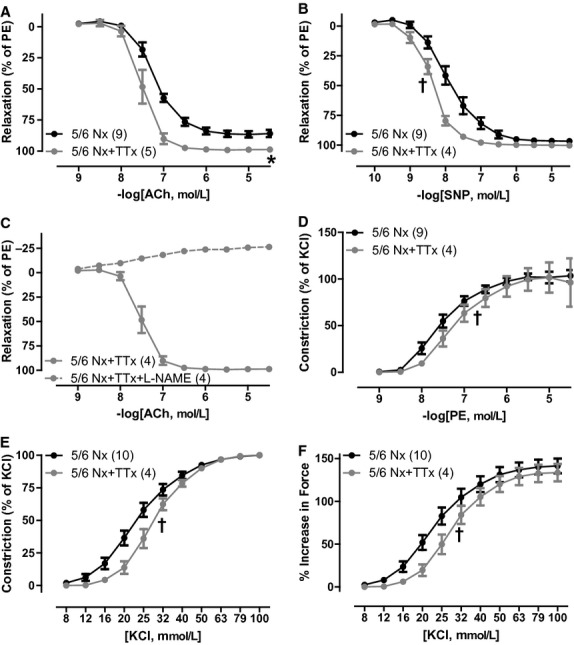
Response of aortas isolated from 5/6 nephrectomized (Nx) rats treated with TTx or vehicle to (A) acetylcholine (ACh), (B) sodium nitroprusside (SNP), (C) TTx effect on dependency of ACh-induced relaxation on NOS in aorta from 5/6 Nx rats was assessed in the presence of the nonspecific NOS inhibitor L-NAME, and (D) phenylephrine (PE). Response to KCl analyzed as constriction (% of KCl) to assess EC_50_ (E) and % increase in force to examine maximum constriction response (F). The number of rats is in parentheses. *P < 0.05 for % maximum relaxation versus non-TTx-treated rats; ^†^*P* < 0.05 for logEC_50_ (or EC_50_ for KCl) versus non-TTx-treated rats.

TTx was effective in reducing mean arterial pressure (MAP) for all 8 weeks of 5/6 Nx (Fig. 3). TTx enhanced endothelial function (E_max_ but not logEC_50_) in aortas from 5/6 Nx rats (Fig. 4A) and SNP response (logEC_50_ but not E_max_) (Fig. 4B). The dependence of ACh-induced relaxation on NOS did not change with TTx treatment (Fig. 4C). Furthermore, TTx treatment blunted sensitivity to PE in aortas from 5/6 Nx rats (Fig. 4D) and also reduced the EC_50_ response to KCl by 1.2-fold (*P* < 0.05) (Fig. 4E), but did not alter the maximum response to KCl when calculated as % increase in force (Fig. 4F).

**Figure 5:**
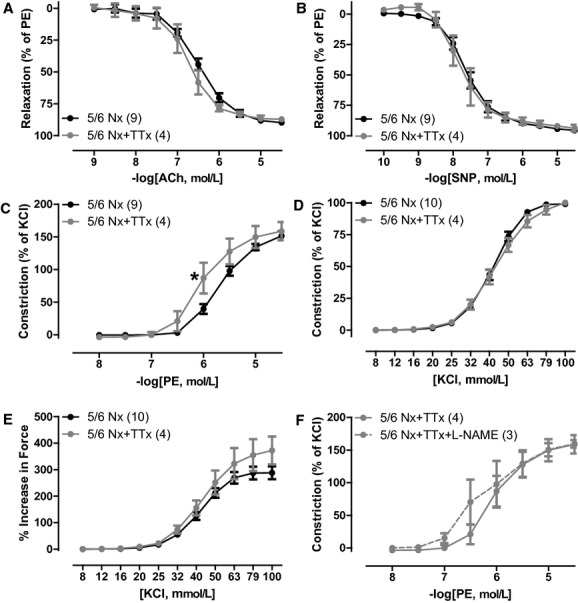
Response of tail arteries isolated from 5/6 nephrectomized (Nx) rats treated with TTx or vehicle to (A) acetylcholine (ACh), (B) sodium nitroprusside (SNP), or (C) phenylephrine (PE). Response to KCl analyzed as constriction (% of KCl) to assess EC_50_ (D) and % increase in force to examine maximum constriction response (E). TTx effect on NOS buffering capacity to PE-induced constriction in tail artery from 5/6 Nx rats was assessed in the presence of the nonspecific NOS inhibitor L-NAME (F). Number of rats is in parentheses. *P < 0.05 for logEC_50_ versus non-TTx-treated rats.

In tail arteries, TTx did not change E_max_ or logEC_50_ responses to ACh in these arteries from 5/6 Nx rats (Fig. 5A) or SNP (Fig. 5B). However, TTx enhanced sensitivity to PE in tail arteries from 5/6 Nx rats (Fig. 5C) without altering the KCl sensitivity (Fig. 5D) or maximum response (Fig. 5E). It was observed in Figure 2E that NOS buffering was enhanced in tail arteries isolated from 5/6 Nx rats. In Figure 5F, nonselective blockade of NOS had no effect on PE-induced constriction in Nx rats on TTx, indicating that elevated MAP in 5/6 Nx rats upregulated NOS buffering in tail arteries to reduce sensitivity to PE-induced constriction.

## Discussion

We observed differential changes in vascular function of central versus peripheral arteries in the setting of CRF. Aortas isolated from 5/6 Nx rats displayed endothelial dysfunction and increased *α*_1_-adrenergic contractility. In contrast, in tail arteries, no change in endothelial function was detected, whereas there was reduced sensitivity to PE in the Nx group. Mechanistically, these changes in vascular function were linked to corresponding changes in NOS function whereby Nx reduced NOS function in aorta but increased NOS function in tail artery. Furthermore, these alterations in NOS function in both aorta and tail artery were linked to the increased blood pressure that accompanied Nx.

Endothelial dysfunction promotes the development of cardiovascular disease, such as atherosclerosis, which contributes to the exceptionally high mortality in patients with CRF (Leskinen et al. 2003; Balla et al. 2013). CRF patients present with endothelial dysfunction (Bolton et al. 2001). Endothelial function is assessed noninvasively in peripheral extremity arteries in humans (Kuvin et al. 2003). However, in animal models, the aorta is commonly used in studies of the vasculature with limited numbers of studies on peripheral arteries. Our current study compared peripheral tail artery and aortic vascular function in a rat model of CRF. We want to highlight that we did not use the rat tail artery to model a resistance vessel. Bessa et al. showed that the diameter of the proximal tail artery, which was used in our study, is approximately 500 *μ*m and is therefore not classified as a resistance artery (<200 *μ*m) (Bessa et al. 2011). Instead, we used the rat tail artery to model a medium-sized artery like those found in the extremities of humans. Indeed, Langewouters et al. (1986) showed that rat tail arteries and human digital arteries share similar vascular reactivity profiles. Because assessment of digital vascular function is an established, noninvasive method used in the clinic, the purpose of our study was to compare the effects of CRF in 5/6 nephrectomized rats on vascular function in tail artery and aorta. We observed endothelial dysfunction in the aorta following 5/6 Nx, whereas tail arteries did not reveal endothelial dysfunction even in the face of hypertension.

Reports of differential function in tail artery and aorta in cardiovascular–renal disease models are scattered throughout the literature without a consensus on the mechanisms responsible (Overbeck and Grissette 1982; Stassen et al. 1997; Brymora et al. 2007). We assessed the vascular NOS function. In the whole animal, intravenous infusion of the nonselective NOS inhibitor L-NAME enhanced blood pressure similarly in healthy and CRF rats (Choi et al. 1997). These data suggest no net change in systemic NOS function. Therefore, it is important to understand the regulation of NOS function in different vascular beds in CRF. Aortas from Nx rats have dramatic endothelial dysfunction along with reduced NOS3 expression (Toba et al. 2011). These data indicate that NOS function is reduced in aortas from Nx rats. Our data support this notion whereby aortic endothelial-dependent vasorelaxation was significantly reduced in Nx rats compared with control rats and L-NAME abolished ACh-mediated relaxation in aortas from both control and Nx rats. Further highlighting reduced NOS function in aortas from Nx rats is the finding that L-NAME enhanced aortic constriction to PE in control rats, but not in the setting of CRF. In contrast to the aorta, tail arteries from Nx rats did not have functional alterations in NOS-mediated vasorelaxation. We did detect a minor amount of relaxation (∼20%) induced by the two highest concentrations of ACh in L-NAME-treated tail arteries from both control and 5/6 Nx rats. Studies by Jia et al. (2002) indicate that this residual vasorelaxation response in tail arteries treated with L-NAME is due to prostacyclin.

Although the L-NAME studies in tail arteries indicated that NOS function in response to ACh was similar between control and 5/6 Nx rats, we observed a differential response with regard to PE-induced constriction. These data indicate that the signaling between the *α*_1_ adrenergic receptor and NOS was enhanced in tail arteries, whereas this response was lost in aortas from hypertensive Nx rats. In our study, L-NAME reversed the blunted sensitivity to PE observed in Nx rats. A link between activation of the *α*_1_ adrenergic receptor and NOS3 was demonstrated in studies performed by Looft-Wilson et al. (2013) where *α*_1_ receptor stimulation using PE led to phosphorylation of NOS3 at the activation site Ser1179.

The observation of improved endothelial function related to hypertension is interesting insofar as it is consistent with emerging evidence that elevated pressure leads to an enhanced role of hydrogen peroxide (H_2_O_2_) as an endothelium-dependent vasodilator. Previous studies from our laboratory have shown that NOS-dependent vasorelaxation is enhanced in response to hypertension. In the setting of angiotensin II-induced hypertension, mesenteric arteries have increased NOS-dependent vasorelaxation even in the presence of endothelial dysfunction (Kang et al. 2007). However, this does not correlate with increased NO production but with increased production of pro-vasodilator, H_2_O_2_. H_2_O_2_ is a vasoactive reactive oxygen species (ROS) that preserves vascular function in hypertension. However, it is established that in hypertensive CRF there is increased aortic superoxide, which is a ROS that can scavenge bioavailable NO (Hasdan et al. 2002). Indeed, we found reduced NOS-mediated vasorelaxation in aortas from rats with CRF that was reversed when hypertension was normalized. We established that the hypertension accompanying 5/6 Nx in rats is responsible for differential changes in aortic and tail artery NOS function. Based on our current findings, we reason that hypertension plays a role in enhancing NOS function in peripheral rat tail arteries possibly via H_2_O_2_.

Additional mechanisms may promote the differential hypertensive effects on aortic and tail artery NOS function in CRF. CRF is linked to increased circulating asymmetric dimethylarginine (ADMA) levels, an endogenous inhibitor of NOS. Matsuguma et al. (2006) confirmed this finding in 5/6 Nx rats and found a significant correlation between plasma ADMA concentration and systolic blood pressure. Interestingly, they also found decreased expression of the ADMA-degrading enzyme *N*^*G*^*,N*^*G*^-dimethylarginine dimethylaminohydrolase (DDAH) as well as increased expression of the ADMA-synthesizing protein methytransferase (PRMT) in whole kidney cortex. These findings demonstrated local tissue production of NOS enzyme regulatory factors in CRF. Investigation of differential regulation of the DDAH/ADMA axis between aorta and tail artery in the context of CRF should be valuable in elucidating the mechanisms linking hypertension and the differential changes in NOS function in various arteries during CRF.

Changes in human digital blood vessel function are sensitive to temperature. The rat tail serves as a heat-loss organ to aid in body temperature regulation (Gordon 1990). The physiology of the phenomenon requires changes in vascular tone in the tail. Specifically, *α* adrenergic receptors mediate constriction with reductions in body temperature, and increases in temperature reduce trafficking of this receptor to the plasma membrane to inhibit constriction-promoting vasodilation. In mechanistic studies, exposure of tail arteries to increased temperature promotes vasodilation mediated by heat-shock protein (HSP) 90 inhibition of *α* adrenergic receptor trafficking to the plasma membrane (Filipeanu et al. 2011). Intriguingly, separate studies have shown that HSP90 is also critical in NOS protein folding and function (Chatterjee et al. 2008; Moleda et al. 2010). We propose that 8 weeks of 5/6 Nx increases NOS function in the peripheral arteries via upregulation of HSP90-dependent mechanism in the face of renal failure.

## Conclusions

Hypertension is common in CRF patients and is a predictor of mortality by promoting the progression of cardiovascular–renal disease (Schiffrin et al. 2007). Human studies indicate that fingertip digital thermal monitoring can predict the degree of cardiovascular disease in patients and that *α* adrenergic receptors mediate constriction in these arteries (Coffman and Cohen 1988; van der Wall et al. 2010). Therefore, it is important to understand how hypertension in the setting of CRF modulates function of these peripheral arteries. Interestingly, pressure-induced diameter changes in human finger arteries and the rat tail artery are similar, which stresses the importance of understanding how rat tail artery function relates to human artery function (Langewouters et al. 1986). Understanding the mechanisms that preserve vascular NOS function in the extremities may lead to preventative therapies to circumvent the need for amputations, which are rampant in CRF patients (O'Hare 2005). Ease-of-access arteries, such as the tail artery and finger vessels, provide compelling new models to assess cardiovascular risk in disease states such as CRF.
